# Bioenergetic reprogramming of articular chondrocytes by exposure to exogenous and endogenous reactive oxygen species and its role in the anabolic response to low oxygen

**DOI:** 10.1002/term.2126

**Published:** 2016-01-22

**Authors:** H. K. Heywood, D. A. Lee

**Affiliations:** ^1^ School of Engineering and Materials Science Queen Mary University of London UK

**Keywords:** chondrocyte, regenerative medicine, reactive oxygen species, mitochondria, antioxidant, *N*‐acetyl cysteine, hypoxia

## Abstract

Monolayer culture is integral to many cell‐based cartilage repair strategies, but chondrocytes lose regenerative potential with increasing duration *in vitro*. This coincides with elevated reactive oxygen species (ROS) levels and a bioenergetic transformation characterized by increasing mitochondrial function. This study investigates ROS as stimuli for bioenergetic reprogramming and the effect of antioxidants on the propensity of chondrocytes to regenerate a cartilaginous matrix. Articular chondrocytes were cultured in monolayer under a 2% O_2_ atmosphere. Oxidative stress was increased using 50 μm H_2_O_2_ or a 20% O_2_ culture atmosphere, or decreased using the antioxidant N‐acetyl‐cysteine (NAC). Mitochondrial function was characterized using 200 nm Mitotracker green and an oxygen biosensor. After two population doublings ± NAC, chondrocytes were encapsulated in alginate beads (1 × 10^7^ cells/ml) for an additional 10 days before DMB assay of glycosaminoglycan content. The beads were cultured under both 20% O_2_ and the more physiological 5% O_2_ condition. Chondrocytes expanded in 20% O_2_ exhibited elevated mitochondrial mass and functional capacity, which was partially mimicked by the exogenous ROS, H_2_O_2_. Oligomycin treatment revealed that the increased oxygen consumption was coupled to oxidative phosphorylation. NAC limited these markers of bioenergetic reprogramming during culture‐expansion with no significant effect on subsequent GAG production under 20% O_2_. However, NAC treatment in monolayer abolished the hypoxic induction of GAG in alginate beads. This supports the hypothesis of a causal relationship between exposure to ROS and acquired mitochondrial function in chondrocytes. Additionally, mitochondrial function may be required for the hypoxic induction of GAG synthesis by chondrocytes. © 2015 The Authors. *Journal of Tissue Engineering and Regenerative Medicine* Published by John Wiley & Sons, Ltd.

## 1 Introduction

Monolayer culture is integral to many cell‐based cartilage repair strategies, but chondrocytes lose regenerative potential with increasing duration *in vitro*. It is well established that during monolayer expansion chondrocytes lose their characteristic morphology and that their expression of key extracellular matrix macromolecules, including sulphated glycosaminoglycans, is diminished (Benya and Shaffer, 1982; Dell'Accio *et al.*, 2001; Giovannini *et al.*, 2010). Chondrocytes also undergo a bioenergetic transformation in culture, switching from an almost exclusively glycolytic energy metabolism towards increasing dependence on mitochondrial oxidative phosphorylation (Champagne *et al.*, 1987; Heywood and Lee, 2008, 2010; Mignotte *et al.*, 1991). There is evidence to suggest that this bioenergetic reprogramming is not readily reversible (Boubriak *et al.*, 2009), potentially persisting on re‐implantation to the joint. Thus, it may be important to understand its cause and consequence to chondrocyte function. This study investigates the stimuli for such bioenergetic reprogramming and its effect on the ability of chondrocytes to regenerate a cartilaginous matrix.

It is well established that chondrocytes exhibit exceptionally low oxygen consumption rates compared to the majority of mammalian cell types (Stockwell, 1991). For example, chondrocyte oxygen consumption rates are reported to be around 1–6 fm/cell/h (Bowie *et al.*, 1941; Heywood *et al.*, 2010; Heywood and Lee, 2008; Rosenthal *et al.*, 1941), compared to approximately 100 fm/cell/h in MSCs (Pattappa *et al.*, 2011) and 325 fm/cell/h in hepatocytes (Balis *et al.*, 1999). Thus, primary chondrocytes derive the majority of their ATP from glycolysis, with estimates attributing just 1–10% of cellular ATP production to mitochondrial oxidative phosphorylation (Heywood *et al.*, 2010, 2014; Heywood and Lee, 2008). However, our laboratory has identified that oxygen consumption increases markedly in chondrocytes that have been cultured in monolayer compared to immediately following isolation (Heywood *et al.*, 2010, 2014; Heywood and Lee, 2008). These studies have confirmed that the additional oxygen consumption in monolayer‐expanded cells is coupled to ATP generation via mitochondrial oxidative phosphorylation and is associated with increased mitochondrial mass (Heywood *et al.*, 2010). Indeed, earlier work by Mignotte and others demonstrated a 13‐fold increase in cellular mitochondrial DNA (mtDNA) relative to total DNA following 6 days of monolayer culture (Champagne *et al.*, 1987; Mignotte *et al.*, 1991).

In addition to the generation of ATP, mitochondria have other roles, including the generation of reactive oxygen species (ROS) as an essential signalling mechanism (Bell *et al.*, 2007a; Cillero‐Pastor *et al.*, 2008; Lee *et al.*, 2000; Milner *et al.*, 2007). There is some evidence to show that sublethal doses of endogenous or exogenous H_2_O_2_ are sufficient to stimulate mitochondrial biogenesis in a number of cell types (Lee and Wei, 2005; Lee *et al.*, 2002, 2000). As such, it has been proposed that ROS act as a stimulus of bioenergetic reprogramming (Lee and Wei, 2005; Venditti *et al.*, 2014). A marked increase in ROS generation and markers of associated oxidative stress are observed by the chondrocytes on transfer into monolayer culture (Heywood and Lee, 2008, 2010; Heywood *et al.*, 2014). However, any causal relationship between elevated ROS *in vitro* and the bioenergetic reprogramming of chondrocytes in monolayer culture has not been examined. Accordingly, this study examines whether: (a) exogenous ROS are sufficient to promote bioenergetic reprogramming in chondrocytes, thereby increasing mitochondrial mass and function; (b) bioenergetic reprogramming can be downregulated by treatment with the pro‐antioxidant, *N*‐acetyl cysteine (NAC); and (c) NAC treatment during monolayer culture affects the subsequent regeneration of a cartilaginous matrix by chondrocytes, with implications for cartilage repair strategies.

## 2 Materials and methods

### 2.1 Cell source

The metacarpophalangeal joints of 18–24 month‐old cattle were opened under sterile conditions. Full‐depth cartilage tissue slices were removed from the proximal joint surface, using a scalpel. Chondrocytes were isolated from the cartilage extracellular matrix by sequential incubation at 37 °C in Dulbecco's modified Eagle's medium (DMEM) with addition of 5.7 mg/ml pronase for 1 h, followed by 100 U/ml collagenase for 14 h, as described previously (Heywood and Lee, 2008). The freshly isolated chondrocytes were seeded into 175 cm^2^ tissue‐culture flasks at a density of 2.2 × 10^4^ cells/cm^2^ and cultured with 40 ml medium/flask at 37 °C in a humidified/5% *v*/*v* CO_2_ incubator atmosphere. Media during culture consisted of DMEM supplemented with 10% *v*/*v* fetal calf serum (FCS), 2 mm l‐glutamine, 20 mm HEPES, 88 U/ml penicillin and 88 mg/ml streptomycin. All reagents were from Sigma‐Aldrich (Poole, UK). Cells from individual donor animals were maintained separately during culture. The joints were obtained as waste material from a commercial abattoir; ethical approval for animals in research was not applicable.

### 2.2 Cell culture

To determine whether exposure to exogenous oxidants can promote an oxidative bioenergetic transformation in culture, cells were cultured under a 2% *v*/*v* O_2_ atmosphere and compared to cells where the level of oxidative stress was increased by either addition of the exogenous oxidant hydrogen peroxide (H_2_O_2_) or increasing the O_2_ atmosphere to 20% *v*/*v* (Heywood and Lee, 2010). An aliquot of H_2_O_2_ was added to selected flasks to provide a final concentration of 50 μm, and replenished at feeding intervals. The oxygen atmosphere was controlled using an XVivo Biospherix (New York) system, with integrated incubators and workspace to maintain a continuous level of O_2_ in the atmosphere during culture and any manipulation. After 9 days, the cells were recovered from the flasks by incubation in trypsin–EDTA solution and resuspended in fresh media. The cell yield was determined by haemocytometer. Aliquots of the cell suspension were used for determination of their bioenergetic phenotype, as defined by mitochondrial mass and key parameters of mitochondrial function, i.e. oxidative phosphorylation, respiratory capacity and ROS, as described below.

**Figure 1:**
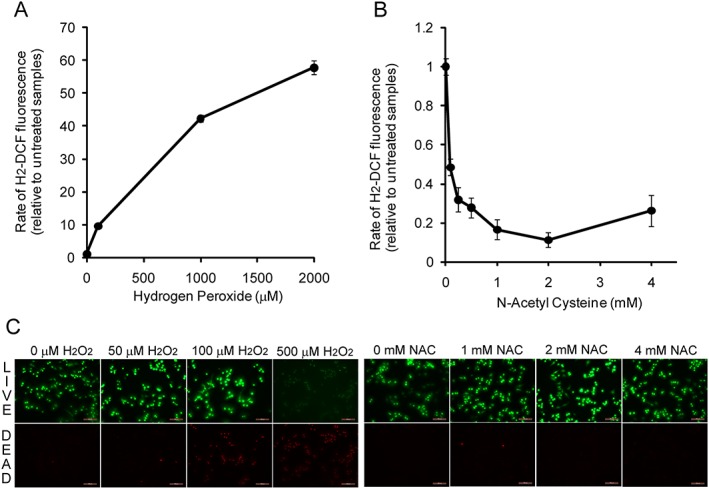
Cellular ROS levels, determined by monitoring the rate of increase in H_2_DCF fluorescence for chondrocytes following exogenous (A) H_2_O_2_ or (B) NAC treatments; data points represent mean ± SE of three and five replicate cell samples for H_2_O_2_ and NAC treatments, respectively. Viability was confirmed in 50 μm H_2_O_2_ and 2 mm NAC by strong cytoplasmic staining in calcein‐AM and few ethidium homodimer‐1 positive nuclei (C) after 24 h of treatment. Scale bar = 100 μm. [Colour figure can be viewed at wileyonlinelibrary.com]

To determine whether reducing oxidative stress inhibits bioenergetic reprogramming, freshly isolated cells were seeded in flasks and cultured under a 20% O_2_ atmosphere in the presence or absence of the pro‐antioxidant *N*‐acetlyl cysteine (NAC). NAC removes cellular H_2_O_2_, a by‐product of superoxide dismutation, by augmenting the activity of the endogenous glutathione antioxidant system (Brand *et al.*, 2004). The probe carboxy‐dichlorodihydrofluoroscein (H_2_DCF; Molecular Probes) becomes fluorescent on oxidation by ROS. A dose–response study in the presence of H_2_DCF revealed that 2 mm NAC reduced cellular ROS levels to <10% of untreated control values, whilst exogenous H_2_O_2_ increased ROS levels, as expected (Figure 1A, B). Viability was maintained in the selected doses of 50 μm H_2_O_2_ and 2 mm NAC, indicated by strong cytoplasmic staining with calcein‐AM (green) and few ethidium homodimer‐1 (red)‐positive nuclei (5 μm; Invitrogen) (Figure 1C). During culture in the presence or absence of NAC, cell proliferation level was monitored by light microscopy and manual cell counting. After a matched level of two population doublings, the culture‐expanded cells were recovered to a suspension for analysis of their bioenergetic phenotype, or encapsulation into alginate to assess their capacity for cartilage matrix regeneration, described below.

### 2.3 Mitochondrial mass and reactive oxygen species

The cells were suspended at 1 × 10^6^ cells/ml and incubated for 1 h at 37 °C in complete DMEM with 200 nm Mitotracker Green (Invitrogen). The cells were washed in warm Hanks' buffer and resuspended at 1 × 10^6^ cells/ml in fresh Hanks' buffer containing 5 μm dihydroethidium (DHE; Invitrogen). 100 μl aliquots were added to a 384‐well assay plate and the fluorescence monitored over 20 min, using 488 nm excitation and 520 nm and 590 nm emission wavelengths to detect mitochondrial mass and ROS, respectively (BMG Optima fluorimeter). An unstained cell suspension controlled for background fluorescence. Addition of xanthine/xanthine oxidase (100 μm/10 μm/ml) was used as a positive control for superoxide generation. DHE solution without cells was used as a negative control to confirm that auto‐oxidation of the dye was negligible.

### 2.4 Oxidative phosphorylation and respiratory capacity

A fluorescence‐based oxygen biosensor (BD Biosciences, UK) was used to monitor the dissolved oxygen concentration in cell suspensions over time, as reported previously (Heywood *et al.*, 2006a, 2010). Briefly, chondrocyte suspensions were prepared in fresh DMEM with cell densities of 2 × 10^6^ cells/ml. 320 μl aliquots of the cell suspensions were loaded into the wells of the 96‐well oxygen biosensor, which was sealed with adhesive film and maintained at 37 °C. The rate of oxygen consumption in the wells was calculated from the gradient of the oxygen–time curve and normalized to total cell number. Oligomycin inhibits the mitochondrial F_1_F_o_ ATP synthase; thus, the proportion of cellular oxygen consumption that was eliminated by the addition of 2 mg/ml oligomycin (Sigma‐Aldrich) is a measure of oxidative phosphorylation. The addition of 3 μm CCCP (Sigma‐Aldrich) uncouples the mitochondrial electron transport chain from ATP demand, revealing changes in the maximal respiratory capacity of the chondrocyte mitochondria.

### 2.5 The effect of NAC treatment on subsequent cartilage matrix regeneration

After two population doublings in monolayer culture, control and NAC‐treated chondrocytes (above) were encapsulated in 2% *w*/*v* alginate, beads as described (Lee *et al.*, 2003), with a density of 10 × 10^6^ cells/ml. The mean volume of the beads was 0.022 ± 0.004 ml (assuming a density of 1 g/ml). The beads were transferred into a 24‐well plate, with three beads and 2 ml medium/well, and cultured for an additional 10 days in fresh medium (± continued NAC treatment), with medium exchange on alternate days. During culture, half the alginate bead samples were maintained under an oxygen atmosphere containing 20% *v*/*v* O_2_, whilst the other half was maintained under a 5% *v*/*v* O_2_ atmosphere, which more closely represents conditions in the joint. The beads were digested in saline sodium citrate buffer with 2.8 U/ml papain and analysed using the DMB assay for the cartilage matrix constituent, sulphated glycosaminoglycan (GAG) with modifications for alginate samples (described fully in Enobakhare *et al.*, 1996).

### 2.6 Statistics

Data presented represent mean and standard error of the mean (SE) from 8 to 18 measurements, derived from three independent experiments, where cells from each donor animal were maintained separately. Statistical comparisons were performed using two‐tailed *t*‐test with Bonferroni correction for multiple comparisons as appropriate. Data for mitochondrial mass were first normalized to represent fold increase from day 0 values.

## 3 Results

Transfer to the *in vitro* environment is associated with increased ROS levels (Heywood and Lee, 2008; Heywood *et al.*, 2014). Here we directly interrogated the role of ROS as a stimulus for bioenergetic reprogramming of chondrocytes.

### 3.1 Exposure to exogenous oxidants promotes an oxidative energy metabolism in chondrocytes

**Figure 2:**
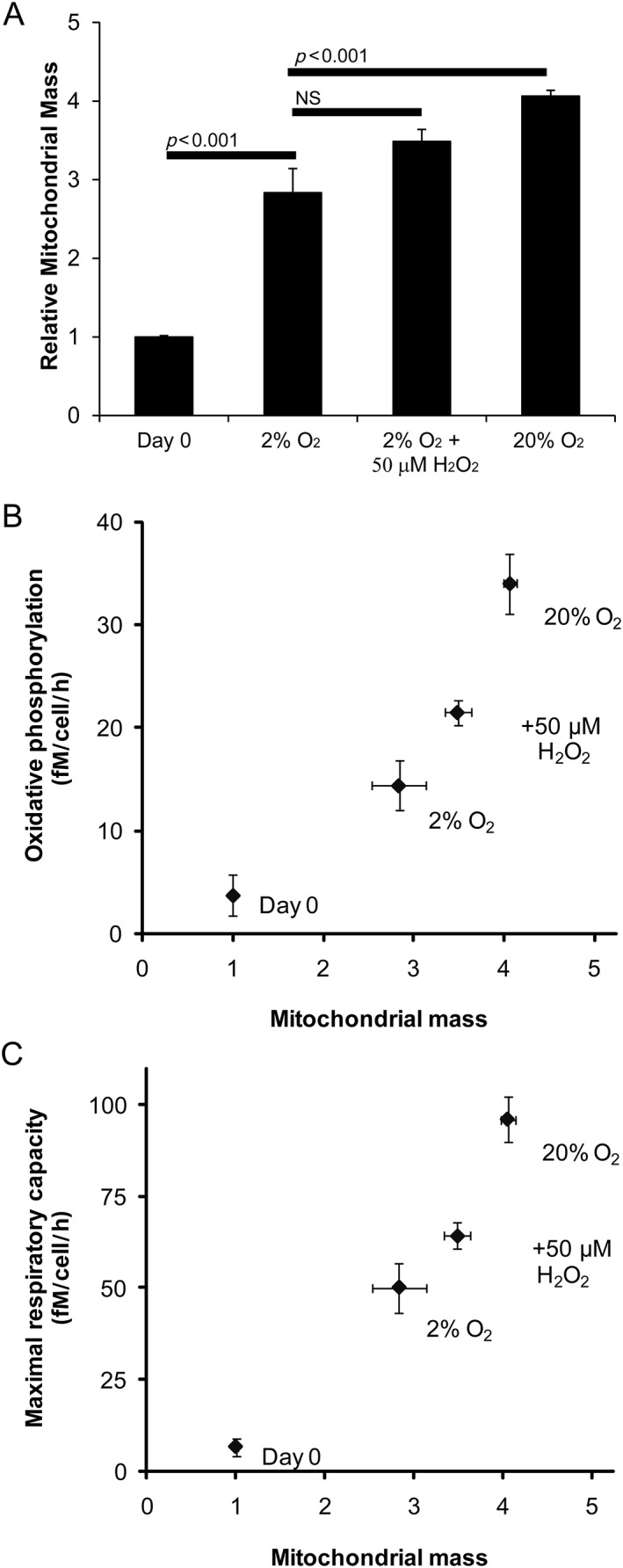
(A) Mitochondrial mass in primary chondrocytes after 9 days in monolayer culture, relative to freshly isolated cells: mitochondrial mass within each treatment group was proportional to key mitochondrial functional parameters, (B) oxidative phosphorylation and (C) and respiratory capacity. Data points represent mean ± SE of 8–18 measurements derived from three experiments

**Figure 3:**
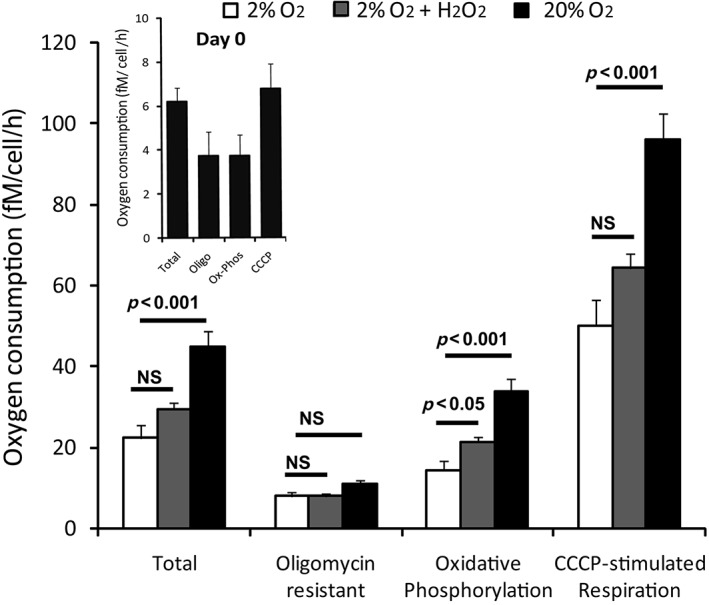
Assessment of oxygen consumption, compartmentalized to indicate mitochondrial functional parameters, for chondrocytes following exposure to exogenous ROS (50 μm H_2_O_2_ or 20% O_2_) during monolayer culture for 9 days, compared to cells cultured at 2% O_2_ and freshly isolated cells (inset). Data represent mean ± SE of eight measurements derived from three experiments

**Figure 4:**
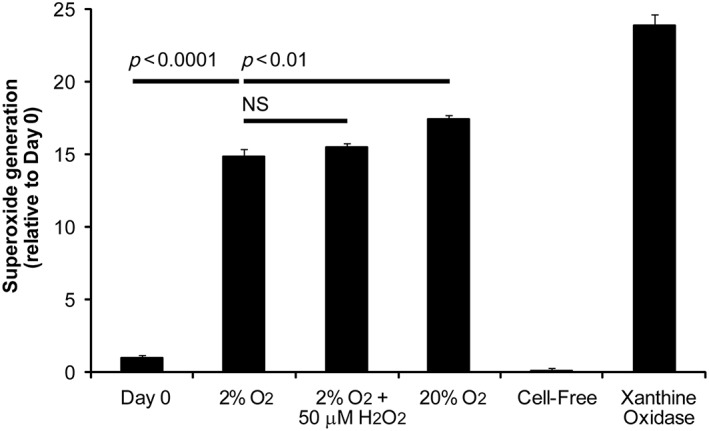
The cellular generation of superoxide, determined by monitoring the rate of increase in dihydroethidium fluorescence over 20 min by chondrocytes following exposure to exogenous ROS (50 μm H_2_O_2_ or 20% O_2_) during monolayer culture for 9 days, compared to cells cultured at 2% O_2_ and freshly isolated cells. Cell‐free and xanthine/xanthine oxidase incubations were included as negative and positive assay controls, respectively. Data represent mean and SE of 8–15 measurements from three independent experiments and are illustrated relative to values obtained for freshly isolated cells

This study aimed to determine whether addition of exogenous ROS was sufficient to promote bioenergetic reprogramming in freshly isolated articular chondrocytes in monolayer. Cells exposed to exogenous ROS by increasing the O_2_ atmosphere to 20% *v*/*v* exhibited significantly increased (*P* < 0.001) mitochondrial mass after 9 days of culture, compared to cells cultured in monolayer under a 2% *v*/*v* O_2_ atmosphere (Figure 2A). Cells exposed to the exogenous ROS, H_2_O_2_, had a similar trend in mitochondrial mass, but this did not reach statistical significance (*P* = 0.06). Greater mitochondrial mass was associated with proportionally increased rates of the mitochondrial functional parameters,oxidative phosphorylation (Figure 2B) and respiratory capacity (Figure 2C). Detailed analysis of the effect of exogenous ROS on chondrocyte mitochondrial metabolism is presented in Figure 3. These data confirm that monolayer cultured cells exhibit a significant (*P* < 0.05) increase in oxygen consumption relative to freshly isolated cells (day 0 values, inset), rising from 6 fm/cell/h to >20 fm/cell/h. Both H_2_O_2_‐ and 20% O_2_‐treated cells exhibited a further significant increase in mitochondrial oxidative phosphorylation compared to the 2% O_2_ condition (*P* < 0.05 and *P* < 0.001, respectively). Culture at 20% O_2_ also significantly increased maximal respiratory capacity recorded in the presence of CCCP (*P* < 0.001), consistent with increased mitochondrial capacity and higher levels of cellular superoxide, as detected by 10 μm dihydroethidium (Figure 4).

### 3.2 Antioxidant treatment down‐modulates the oxidative metabolic transformation

**Figure 5:**
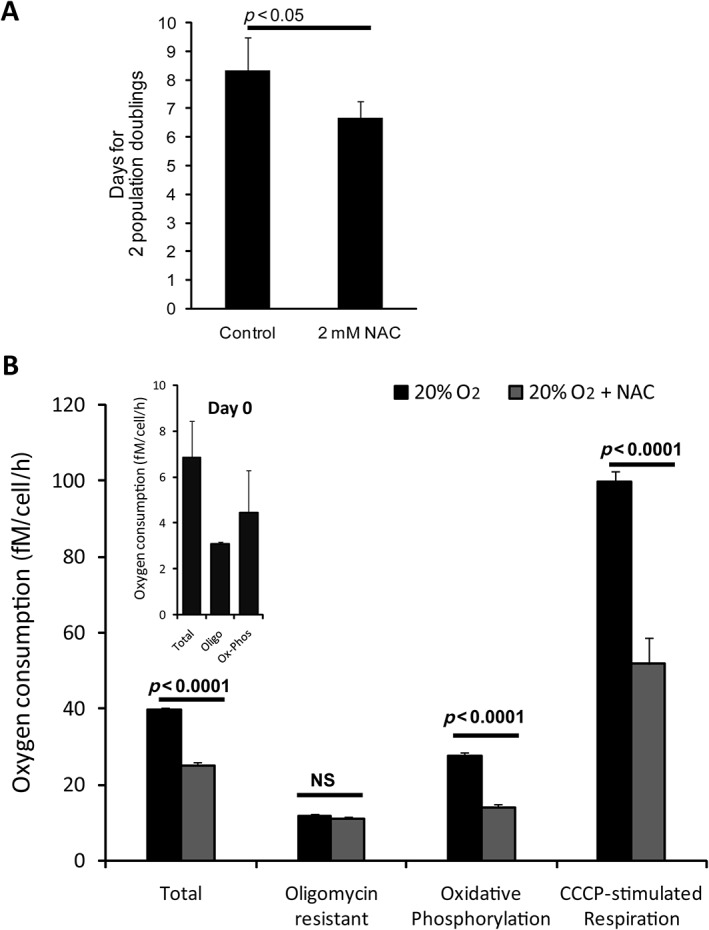
(A) The duration of monolayer culture required for freshly isolated chondrocytes to reach two population doublings for cells cultured at 20% O_2_ in the presence or absence of 2 mm NAC; data represent mean and SD of three measurements, each representing cells from an individual donor animal. (B) Oxygen consumption, compartmentalized to indicate mitochondrial functional parameters for chondrocytes following monoloyer expansion at 20% O_2_ to two population doublings in the presence or absence of 2 mm NAC, compared to freshly isolated cells (inset); data represent mean ± SE of eight measurements derived from three experiments

This study examined whether reducing endogenous ROS could block the oxidative metabolic transformation observed when primary chondrocytes are cultured *in vitro*. Treatment with 2 mm NAC significantly enhanced the rate of proliferation of the cells during expansion in monolayer culture, resulting in a lower population‐doubling time (Figure 5A). Accordingly, measurement of metabolic parameters was performed at a matched proliferation level of two population doublings. Treatment with 2 mm NAC significantly reduced the oxidative metabolic transformation during culture (Figure 5B). Oxidative phosphorylation was significantly reduced (*P* < 0.0001), resulting in lower total oxygen consumption rates. The NAC‐treated cells also exhibited reduced respiratory capacity, determined from CCCP‐stimulated respiration (*P* < 0.0001), which is consistent with lower mitochondrial mass.

### 3.3 The effect of *N*‐acetyl cysteine treatment on the subsequent regenerative activity of articular chondrocytes in 3D culture

**Figure 6:**
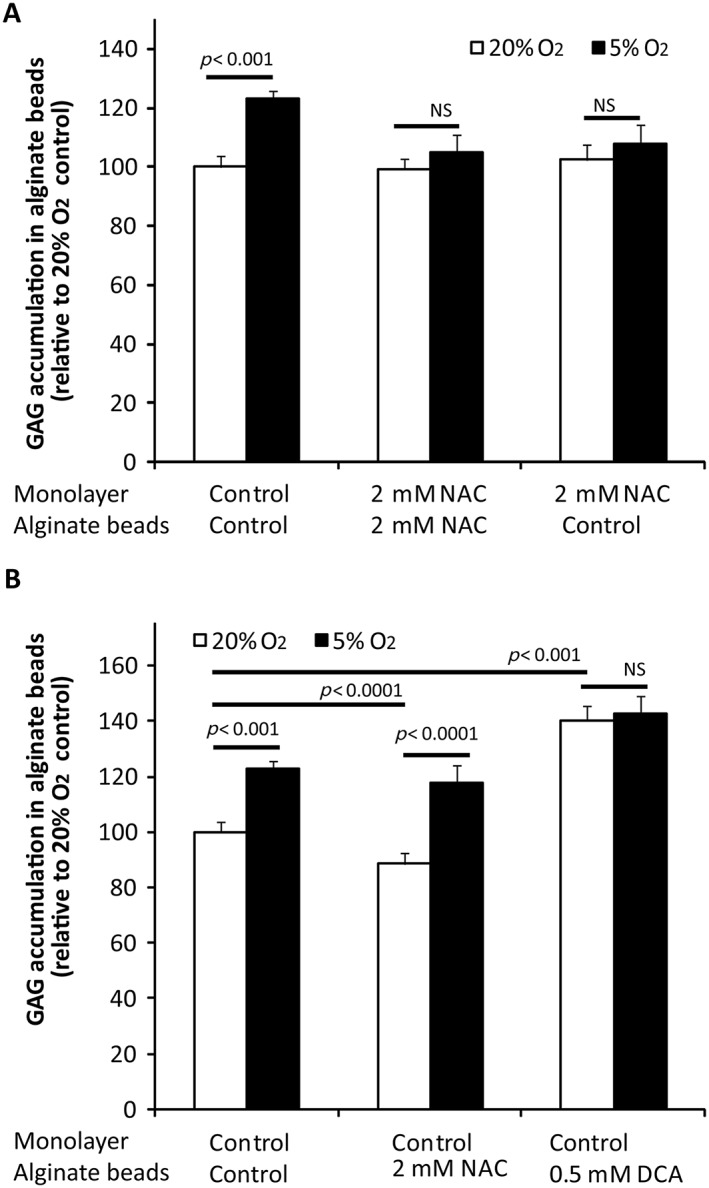
The accumulation of sulphated glycosaminoglycan (GAG) by monolayer‐expanded chondrocytes following 10 days of culture in alginate beads; effect of 2 mm NAC treatment during (A) the monolayer phase or (B) the alginate phase only on GAG accumulation. Alginate beads were cultured under either 5% or 20% O_2_ to assess the anabolic response to hypoxia. The final GAG concentration of alginate beads seeded with untreated cells and cultured under normoxic conditions (to which other samples were normalized) was 1.04 μg/mg. Data represent mean ± SE of 18 alginate beads from three independent experiments; 0.5 mm dichloroacetate (DCA) was used to stimulate mitochondrial function

The ability of monolayer‐cultured chondrocytes to regenerate a cartilaginous matrix once re‐implanted into a cartilage defect is vital to the success of cell‐based repair strategies. In this study, monolayer‐expanded chondrocytes were encapsulated within alginate beads in order to assess the effect of NAC treatment during population expansion on the subsequent propensity of the cells, when embedded in a 3D culture environment, to synthesize sulphated GAG, a key component of the cartilaginous extracellular matrix. Alginate beads seeded with untreated cells accumulated 23% more GAG under 5% O_2_ compared to 20% O_2_ (Figure 6A). Antioxidant treatment during culture–expansion had no significant effect on subsequent GAG accumulation in alginate beads cultured under 20% O_2_. However, NAC treatment during monolayer culture resulted in reduced GAG levels in beads cultured under 5% O_2_ compared to their untreated counterparts. Thus, it appeared that prior treatment in monolayer with NAC may impair the subsequent hypoxic induction of GAG in the alginate beads. This observation persisted even when cells treated in monolayer with NAC were washed before culturing in control media during the alginate phase (Figure 6A).

This phenomenon was investigated further by examining the effect of adding NAC treatment to reduce ROS during the alginate culture phase only. Here, the cells retained a hypoxic response, demonstrated as increased GAG accumulation during culture at 5% O_2_ (Figure 6B). This suggests that the previously observed effect of NAC to block this response may have been acquired only during the monolayer phase, concurrent with the suppressive effect of NAC on the acquisition of mitochondrial function in monolayer. To explore the role of acquired mitochondrial function for the induction of GAG further, additional cell samples were treated with 0.5 mm dichloroacetate (DCA). As a pyruvate dehydrogenase activator, dichloroacetate rapidly stimulates mitochondrial function (Michelakis *et al.*, 2008). Dichloroacetate treatment significantly increased GAG accumulation in alginate beads by >40% compared to untreated control cells (Figure 6B), which could not be increased further by reducing the oxygen level to 5% *v*/*v*. Thus, mitochondria mediate the anabolic response of culture‐expanded chondrocytes to reduced oxygen levels.

## 4 Discussion

Many cell‐based cartilage repair strategies utilize an *in vitro* culture phase for population expansion. There is evidence indicating that the acquisition of a more aerobic energy metabolism *in vitro* may not be readily reversed on re‐introduction to 3D culture (Boubriak *et al.*, 2009), and so it is important to understand both the mechanisms and potential consequences of such bioenergetic reprogramming to tissue repair. This study supports the hypothesis that bioenergetic reprogramming of chondrocytes in monolayer can be modulated by ROS, which are a natural by‐product of mitochondrial activity. Up to 5% of total O_2_ consumption is channelled into superoxide production in the mitochondrial electron transport chain (Boveris *et al.*, 1972; Turrens, 2003). Superoxide is predominantly generated at complexes I and III of the electron transport chain and is subsequently subject to dismutation into hydrogen peroxide (H_2_O_2_), which may then be removed by the glutathione antioxidant system (Brand *et al.*, 2004). The aerobic environment during monolayer culture *in vitro* is anticipated to increase the production of superoxide in chondrocyte mitochondria, by the mass action of oxygen on the electron transport chain (Boveris and Chance, 1973; Turrens, 2003). As such, it has been proposed that ROS act as a potential stimulus of bioenergetic reprogramming which may participate in a positive feedback cycle, promoting increased oxygen consumption and further increases in ROS.

Monolayer culture under 20% O_2_ increased parameters of mitochondrial function, including mitochondrial mass, oxidative phosphorylation and maximal respiratory capacity, consistent with the literature (Champagne *et al.*, 1987; Heywood and Lee, 2008, 2010; Mignotte *et al.*, 1991). This effect was substantially reduced by culture at 2% O_2_. Treatment of cells cultured under 20% O_2_ with the pro‐antioxidant NAC substantially reduced alteration in mitochondrial function to levels similar to those observed in cells cultured at 2% O_2_. Moreover the effect of culture at 20% O_2_ could be partially mimicked by treatment of cells at 2% O_2_ with the exogenous ROS, H_2_O_2_. These findings are consistent with observations from other cells types (Lee *et al.*, 2000; Lee and Wei, 2005), but is notable because *in vivo* chondrocytes have an exceptionally low mitochondrial density (Brighton *et al.*, 1984; Champagne *et al.*, 1987).

A key finding of this study is that treatment with NAC helped to maintain a metabolic phenotype more comparable to that of primary chondrocytes by reducing the shift towards an aerobic energy metabolism. Moreover, cells cultured in the presence of NAC required significantly less time to reach two population doublings. Accelerated proliferation kinetics is potentially beneficial to cell‐based cartilage therapies, such as autologous chondrocyte implantation, which typically require that chondrocytes are cultured *in vitro* until three or four population doublings are achieved. Accelerating this phase could provide significant savings to the overall treatment times and procedure costs. However, any such benefits will only be realized if the propensity to regenerate a cartilaginous matrix on re‐implantation to the joint is not impaired by prior NAC treatment.

The ability of monolayer‐expanded chondrocytes to regenerate a cartilaginous matrix was assessed on re‐introduction to 3D culture conditions. Only the GAG accumulating in the beads was determined here, representing the GAG that ultimately contributes to new tissue formation. It is possible that an altered proportion of GAG loss to the media between treatment groups may occur under some circumstances. However, we note that previous studies which have examined GAG retention found that the proportion of GAG lost to the culture medium is unaffected by treatment of normal chondrocytes with NAC, hypoxia or glucose level (Collins *et al.*, 2015; Markway *et al.*, 2013; Heywood *et al.*, 2006b).

Once encapsulated into alginate beads and cultured under a 20% O_2_ atmosphere, chondrocytes expanded in monolayer in the presence of NAC accumulated similar quantities of the cartilage extracellular matrix constituent GAG, compared to untreated cells. This is consistent with the absence of any significant effect of NAC treatment on the expression of cartilage phenotype markers, SOX9, type II collagen and aggrecan, immediately prior to alginate encapsulation (see supporting information, Figure S1A and Table S1). Induction of hypertrophy is also associated with altered capacity for articular cartilage repair and may be induced by elevated ROS levels (Kishimoto *et al.*, 2010; Morita *et al.*, 2007). However, no significant difference in the expression of hypertrophic markers, collagen type X and Activin receptor‐like kinase‐1 (Dell'Accio *et al.*, 2001; Markway *et al.*, 2013; van den Berg, 2011) were observed after expansion with NAC (see supporting information, Figure S1B). Accordingly, GAG accumulation under normoxic conditions was neither augmented nor fundamentally impaired by NAC treatment during the earlier expansion phase. However, conditions in the joint are expected to be 1–10% O_2_ (Grimshaw and Mason, 2000), and reducing the O_2_ concentration within this range is reported to increase chondrocyte matrix synthesis, including GAG accumulation (Domm *et al.*, 2002; Lafont *et al.*, 2008; Li *et al.*, 2014; Murphy and Polak, 2004; Murphy and Sambanis, 2001). This effect was observed in the current study, with an enhancement of GAG accumulation for chondrocytes expanded in monolayer at 20% O_2_ when oxygen in the incubator atmosphere was reduced to 5% *v*/*v* during the subsequent culture within alginate beads (Figure 6). The presence of NAC during monolayer expansion abolished this anabolic response to hypoxia during subsequent alginate culture. This highlights a potential detrimental effect on the regenerative behaviour of cells expanded with NAC on re‐implantation into a cartilage defect under low oxygen conditions. Interestingly, the anabolic response to hypoxia was retained when NAC was provided only during the alginate culture phase but was absent during monolayer expansion.

Mitochondrial function is important for hypoxia‐signalling mechanisms in mammalian cells. Although the precise mechanisms are unclear, it has been reported that ROS produced under hypoxia by the mitochondrial electron transport chain are involved in the stabilization of the hypoxic inducible factor‐1*α* subunit (HIF1‐*α*) protein (Ball *et al.*, 2012; Bell *et al.*, 2007a, 2007b), a master‐regulator of hypoxic signalling. However, others report that altered respiratory activity of the mitochondria modulates the cellular hypoxic response by acting as an oxygen sink, which decreases the local cellular oxygen concentration below a critical threshold (Chua *et al.*, 2010; Li *et al.*, 2014). The observation that NAC treatment only blocked the hypoxic induction of GAG when applied during the monolayer phase, and not the alginate phase alone, supports the second hypothesis. This was further supported by the augmentation of GAG accumulation under 20% O_2_ conditions by the mitochondrial activator dichloroacetate (Figure 6). Together, these data raise the intriguing concept that the acquired mitochondrial function may play an important role in mediating the anabolic response of culture‐expanded chondrocytes to reduced oxygen levels.

In summary, the current study supports the hypothesis of a causal relationship between exposure to ROS and bioenergetic reprogramming in articular chondrocytes. Additionally, mitochondrial function may be required for the hypoxic induction of GAG synthesis by cultured chondrocytes. This reveals that bioenergetic reprogramming *in vitro* may influence chondrocyte function following re‐implantation *in vivo*. The antioxidant NAC inhibited the acquisition of mitochondrial function in monolayer. Although these chondrocytes required less time to reach a designated proliferation level, this potential benefit to cell‐based cartilage therapies is likely to be outweighed by the concurrent impairment of cartilage matrix regeneration that was observed on re‐implantation to a hypoxic environment such as the joint.

## Conflict of interest

The authors declare no conflicts of interest.

## Supporting information

Figure S1. Gene expression (A) considered representative of cartilage phenotype (SOX9, collagen type II and aggrecan) or (B) associated with cartilage hypertrophy (collagen type X and Activin receptor‐like kinase receptor‐1) were assessed. Total mRNA of monolayer cells cultured for 2 population doublings under 20% O2 in the presence or absence of 2 mM NAC was extracted and prepared for qPCR analysis of gene expression as described in (Heywood et al., 2014). No significant differences were observed (paired t‐test) between control and NAC cultured cells with either phenotypic or hypertrophic markers. Data represents the mean ± SD of 3 cell donors, tested in duplicate and additional replicate qPCR reactions. Primer sequences are given in Table S1. Gene expression was assessed using a standard curve prepared by serial dilution of cDNA of freshly isolated chondrocytes with normalization to β2 microglobulin housekeeping control. COL10A1 and ALK‐1 are expected to have lower expression in freshly isolated cells and therefore the standards were prepared by dilution series of a preliminary PCR amplification.
Table S1. Sequences of primers used for qPCR to examine expression of phenotypic and hypertrophic genes in chondrocytes.

Supporting info item
