# Supplementary material for: Bioenergetic reprogramming of articular chondrocytes by exposure to exogenous and endogenous reactive oxygen species and its role in the anabolic response to low oxygen
Source: J Tissue Eng Regen Med. 2016 Jan 22;11(8):2286–94. doi: 10.1002/term.2126 (PMC5172424; doi:10.1002/term.2126)
Supplement: Supplementary file 1 — Supporting info item [file TERM-11-2286-s001.zip › Supplementary Table 1_Primer Sequences.docx]

**Supplementary table 1: Sequences of primers used for qPCR to examine expression of phenotypic and hypertrophic genes in chondrocytes.**

| **Genes** | **Sense** | **Anti-sense** | **Annealing temperature** |
| --- | --- | --- | --- |
| COL2A1 | GCAAGAGGCCCCTGCAGGTG | AGCACAAAGCACAAGCCAGTATGT | 60 °C |
| Aggrecan | GATGCTTCTATCCCAGCCTCCGC | CGGTCCGGGAAGTGGCGGTAA | 60°C |
| SOX9 | ACTCTGGGCAAGCTCTGGAGACT | GGCGCGGCTGGTACTTGTAGTCC | 60°C |
| COL10A1 | AAAGGTCTAAGTGGCCCCTTTTGTC | GAGGTTCATGACAAAAGCACCTTGC | 60°C |
| ALK-1 | TGAACCCAAGGAGTCTTGC | GCCTCCTTTTTGTCGCATAG | 55°C |
| β2 Microglobulin | GGGTGCTACATGTCCATGTTTGACC | TGCAGAAGACACCCAGATGTTGATG | 60°C |

*COL2A1, Collagen type II; COL10A1, Collagen type X; ALk-1, Activin receptor-like kinase receptor-1.*
