# Supplementary figures and images for: Bioenergetic reprogramming of articular chondrocytes by exposure to exogenous and endogenous reactive oxygen species and its role in the anabolic response to low oxygen
Source: J Tissue Eng Regen Med. 2016 Jan 22;11(8):2286–94. doi: 10.1002/term.2126 (PMC5172424; doi:10.1002/term.2126)

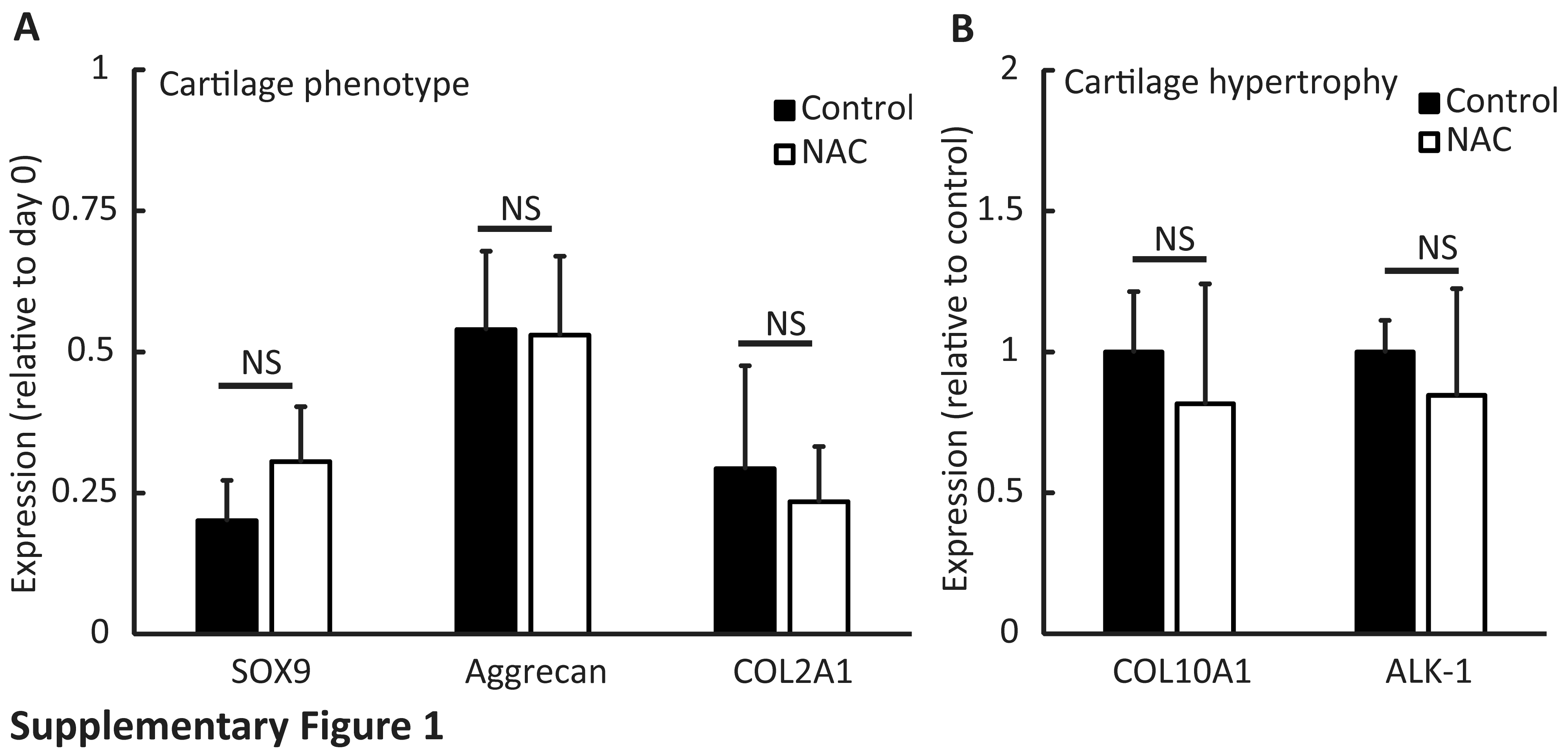

Supplement: Supplementary file 1 — Supporting info item [file TERM-11-2286-s001.zip › Supplementary Figure 1.tif]
